# Simple regression for correcting ΔC_t_ bias in RT-qPCR low-density array data normalization

**DOI:** 10.1186/s12864-015-1274-1

**Published:** 2015-02-14

**Authors:** Xiangqin Cui, Shaohua Yu, Ashutosh Tamhane, Zenoria L Causey, Adam Steg, Maria I Danila, Richard J Reynolds, Jinyi Wang, Keith C Wanzeck, Qi Tang, Stephanie S Ledbetter, David T Redden, Martin R Johnson, S Louis Bridges

**Affiliations:** Department of Biostatistics, School of Public Health, University of Alabama at Birmingham, Birmingham, AL 35294 USA; Division of Clinical Immunology and Rheumatology, University of Alabama at Birmingham, Birmingham, AL 35294 USA; Department of Pharmacology and Toxicology, University of Alabama at Birmingham, Birmingham, AL 35294 USA; Department of Rheumatology and Immunology, Second Xiangya Hospital of Central South University, Changsha, P. R. China

**Keywords:** RT-PCR, Normalization, ΔC_t_, Housekeeping genes, Regression

## Abstract

**Background:**

Reverse transcription quantitative PCR (RT-qPCR) is considered the gold standard for quantifying relative gene expression. Normalization of RT-qPCR data is commonly achieved by subtracting the C_t_ values of the internal reference genes from the C_t_ values of the target genes to obtain ΔC_t_. ΔC_t_ values are then used to derive ΔΔC_t_ when compared to a control group or to conduct further statistical analysis.

**Results:**

We examined two rheumatoid arthritis RT-qPCR low density array datasets and found that this normalization method introduces substantial bias due to differences in PCR amplification efficiency among genes. This bias results in undesirable correlations between target genes and reference genes, which affect the estimation of fold changes and the tests for differentially expressed genes. Similar biases were also found in multiple public mRNA and miRNA RT-qPCR array datasets we analysed. We propose to regress the C_t_ values of the target genes onto those of the reference genes to obtain regression coefficients, which are then used to adjust the reference gene C_t_ values before calculating ΔC_t_.

**Conclusions:**

The per-gene regression method effectively removes the ΔC_t_ bias. This method can be applied to both low density RT-qPCR arrays and individual RT-qPCR assays.

**Electronic supplementary material:**

The online version of this article (doi:10.1186/s12864-015-1274-1) contains supplementary material, which is available to authorized users.

## Background

Reverse transcription quantitative PCR (RT-qPCR) has long become the gold standard for quantifying relative gene expression to study normal and pathological cell processes. Low density RT-qPCR arrays improve the throughput without losing the benefit of individual PCR reactions [1-3]. Although some data-driven normalization methods, such as quantile [4] and rank invariant [5] procedures, have been proposed and applied [6], the most common practice is based on the endogenous internal references, often referred to as “housekeeping” genes as for individual RT-qPCR experiments. Comparison to reference genes offers multiple practical advantages but the use of this strategy relies on the premise that these genes are expressed at the same level across a number of experimental conditions under investigation. However, no endogenous controls have been found to be constantly expressed across all different tissues, developmental stages, and study conditions [7,8]. Thus, a large number of papers focus on identifying stable references for various organisms, tissues, and conditions [9-16], given the critical nature of the quality of the comparisons and the implications for hypothesis testing of expression levels.

Conventional normalization of RT-qPCR data entails first identifying the appropriate reference genes, then subtracting the C_t_ (threshold cycle) values of the best reference gene or the C_t_ mean of several reference genes from all the target genes to obtain the normalized (calibrated) ΔC_t_ for further comparison [17,18]. This type of normalization is based on the assumption that the C_t_ values of the target genes have a linear relationship with those of the reference genes and that the regression coefficient is 1. In this paper, we show, with RT-qPCR array data collected from rheumatoid arthritis patients, that the relationship is linear but the coefficient is not 1 and varies among different reference genes. Under this circumstance, ΔC_t_ is biased. Using a variety of publicly available datasets, we show that this bias is widespread and not related to the physiologic or pathologic process under analysis. Furthermore, we demonstrate that PCR amplification efficiency varies substantially across genes, which is likely the cause of this bias. Methods have been proposed to take into account of the amplification efficiency in the normalization [19,20]; however, they involve estimating amplification efficiencies of targets and references using dilution series, which is not practical for RT-qPCR arrays. We propose a simple regression method for removing ΔC_t_ bias. This method can be applied not only to RT-qPCR arrays but also assays for individual genes.

## Results

### ΔC_t_ normalization introduces bias

**Figure 1:**
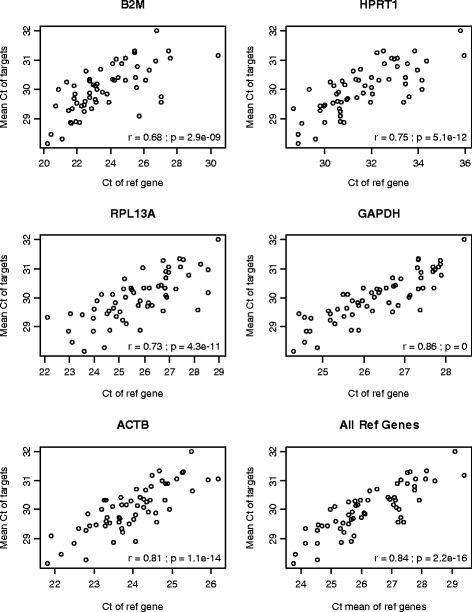
**Mean C**_**t**_ **values of the target genes from each sample are positively correlated with the C**_**t**_ **values of the reference genes on the array.** Results are shown from the rheumatoid arthritis SAB dataset. The lower right panel is based on the C_t_ means of all five reference genes while the others are based on individual reference gene. Ref, reference; r, Pearson correlation coefficient; p, p value from testing the correlation coefficient against 0.

**Figure 2:**
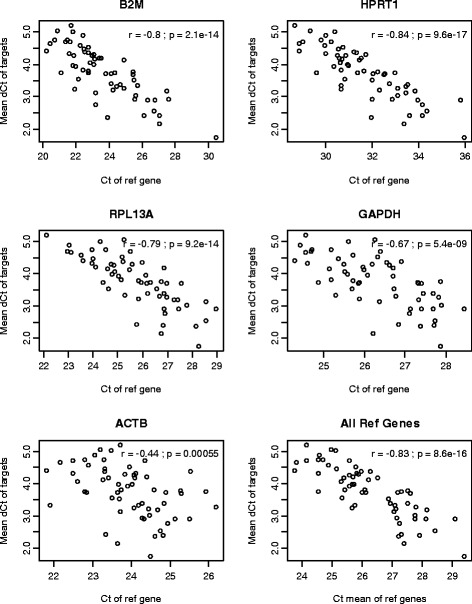
**Negative correlation between the mean of the ΔC**_**t**_ **values from the target genes and C**_**t**_ **values of the reference genes after normalization via conventional subtraction.** The lower right panel is based the C_t_ mean of all five reference genes while the others are based on individual reference gene. Ref, reference; dCt, ΔC_t_; r, Pearson correlation coefficient; p, p value from testing the correlation coefficient against 0.

The commonly used normalization method for RT-qPCR data is subtracting the C_t_ values of the internal reference genes from those of the target genes to obtain the difference in the C_t_ (ΔC_t_). The premise is that differences in the loading amount of template would be represented by the different C_t_ values of the reference genes. Therefore, subtracting the C_t_ of the reference genes (or taking the ratio on the exponential scale) would adjust for these RNA loading differences. To assess the validity of this premise, we plotted the mean C_t_ values of the target genes from a low-density PCR-based array (SAB array), which represent the average signal strength of the target genes, against the reference gene C_t_ values. If the premise were correct, there would be a positive correlation. As expected, the mean C_t_ values of the target genes were indeed positively correlated (r between 0.68 and 0.86) with the C_t_ values of the reference genes (Figure 1). However, after subtracting the reference gene C_t_ values, a negative correlation (r between −0.84 to −0.44) was generated between the mean of the ΔC_t_ values of the target genes and the C_t_ values of each reference gene (Figure 2). This finding indicates a systematic over-correction (bias). If there were no bias, there would be no significant correlation between the mean ΔC_t_ values of the target genes and the reference gene C_t_ values. All five reference genes showed similar negative correlation although the degree varied, which indicates that this is a general phenomenon instead of the property of a particular reference gene. The negative correlation remained present (r = −0.83) when the geometric mean of multiple reference genes (instead of individual reference genes) was used (Figure 2).

### Regression on reference genes

**Figure 3:**
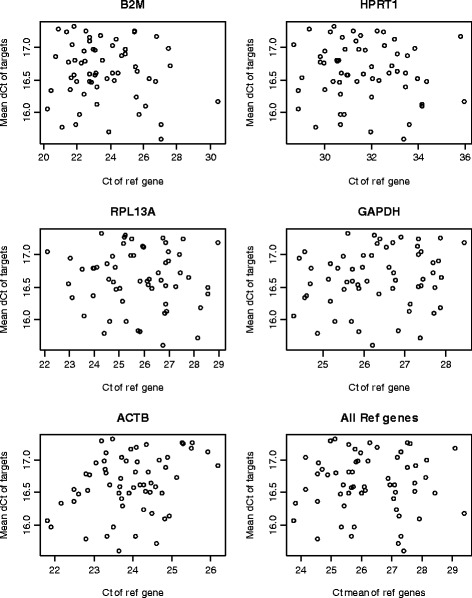
**Multiple regression based normalization removes dependency of target C**_**t**_ **means on reference genes.** No obvious correlation is observed between normalized target gene C_t_ means and C_t_ values of reference genes.

The negative correlation bias shown in Figure 2 indicates that the target genes measurements are linearly related to the reference genes but the coefficients are less than one. When direct subtraction is used, a negative relationship is generated from over-correction. A simple way to solve this problem is to run a linear regression to estimate a coefficient and then adjust the reference gene C_t_ values with the estimated coefficient. Regression analysis can be performed either on any selected individual reference gene or on the mean C_t_ values of all reference genes. The latter approach has the advantage of minimizing the potential undesirable effect of a single reference gene. However, a more comprehensive method is to run a multiple regression including all the reference genes to estimate coefficients for each of them and remove the dependency together (Figure 3). This multiple regression approach is feasible when the number of samples is sufficient (60 in the RA datasets); otherwise, there is the risk of model over-fitting.

### Similar bias from other mRNA RT-qPCR array datasets

To assess whether the ΔC_t_ bias exists with other PCR-based mRNA array datasets, we examined another of our datasets (RA ABI dataset) generated from a different array as well as three publicly available datasets from Gene Expression Omnibus (GEO). Table 1 shows the regression coefficients of the reference genes from these datasets against the mean target C_t_ values from each sample. The coefficients range from 0.19 to 1.09 but are generally less than 1. It is interesting that different experiments show quite different coefficients even for the same reference gene, which necessitated the estimation of regression coefficients in each experiment. For coefficients close to 1, ΔC_t_ does not generate much bias, but for the coefficients far from 1, the bias can be substantial.

**Table 1:** **Reference gene regression coefficients from gene expression datasets**

**Genes**	**GSE15488 GPL8370**	**GSE11690 GPL6933**	**GSET11690 GPL6926**	**RA-SAB**	**RA-ABI**
B2M	0.6782	0.8999	1.0480	0. 2622	NA
HPRT1	0.8605	0.6618	0.1907	0.3598	NA
HPL13A	0.9259	0.9004	0.6230	0.3835	NA
GAPDH	0.8820	0.8054	0.7669	0.6427	0.4720
ACTB	0.6166	0.9611	1.093	0.6831	0.4405
18S	NA	NA	NA	NA	0.0783
GUSB	NA	NA	NA	NA	0.5834
PGK1	NA	NA	NA	NA	0.5607
TFRC	NA	NA	NA	NA	0.3861

NA, not assayed.

### Similar bias from microRNA PCR array datasets

RT-qPCR based low-density arrays are also widely used to assay the expression of microRNA (miRNA). Internal controls built on the array, such as RNU44 and RNU48, are similar to the reference genes on the low-density mRNA arrays. There is evidence that normalizing against the global mean is better than against internal controls for miRNA array data [21,22]. However, the majority of studies still rely on internal controls for normalization. We analyzed four publicly available datasets for regression coefficients in the same fashion as for the mRNA datasets. The results showed that the coefficients for the internal controls are even smaller (Table 2) than those from the mRNA RT-qPCR array datasets. Therefore, the bias resulted from ΔC_t_ normalization would be even more prominent.

**Table 2:** **Control gene regression coefficients from microRNA datasets**

**Controls**	**GSE19229 GPL9732**	**GSE2264 GPL10522**	**GSE39105 GPL15765**	**GSE25868 GPL11239**
MammU6	0.3733	NA	NA	NA
RNU44	0.3143	0.4924	0.0515	NL
RNU48	0.3096	0.4578	0.1450	NL
RNU43	NA	NA	0.3554	NA
RNU49	NA	NA	0.2981	NA
RNU6B	NA	0.6446	NA	NA

NA, not assayed. NL, no significant linear relationship.

### Regression coefficients vary among target genes

**Figure 4:**
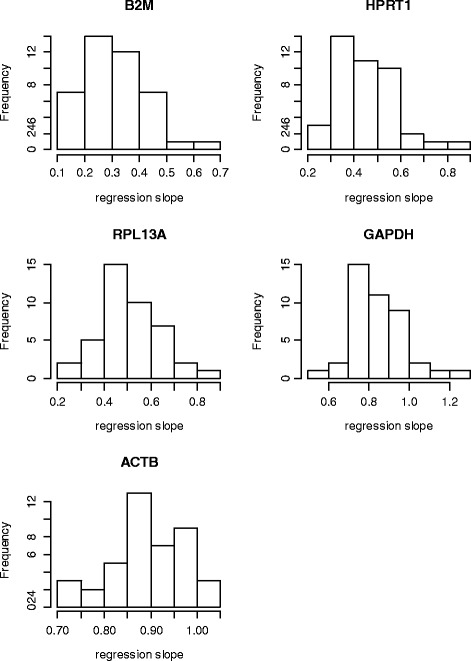
**Histograms of single-gene regression coefficients (slopes) of target gene C**_**t**_ **values on mean reference C**_**t**_ **values.**

To this point, our analyses used the C_t_ means of all target genes on the array from each sample for examining the relationship to the reference genes. When individual target genes were examined, their C_t_ values all showed positive correlations with the reference genes but their regression coefficients varied widely (Figure 4 and Additional file 1: Figure S1.). Only a small number of genes had coefficients close to 1, in which case ΔC_t_ is not biased. The majority of the target genes have coefficients substantially smaller than 1, for which bias will be introduced from the direct subtraction of reference gene C_t_ values in the ΔC_t_ normalization.

### Amplification efficiencies differ among genes

**Figure 5:**
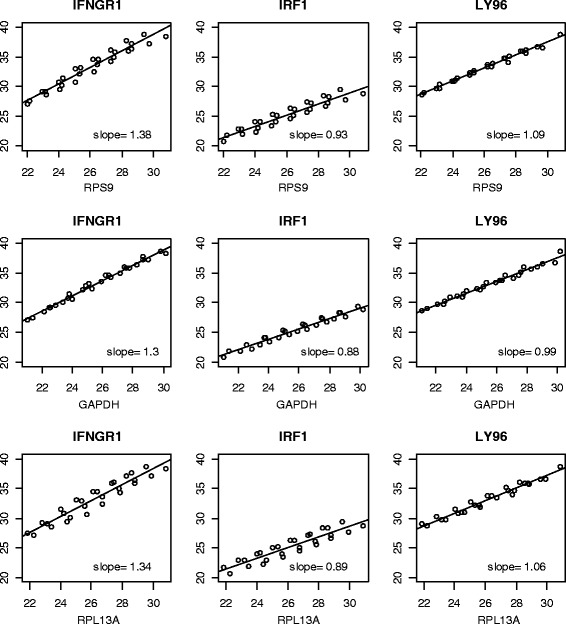
**Regression coefficients between targets and references vary based on the amplification efficiency of both target and references.** The dilution series from all four samples were used to obtain the regression coefficient for each pair of target and reference control.

The deviation of the regression coefficients from 1 is very likely due to amplification efficiency differences between target and reference genes. To check the amplification efficiency, we selected 6 genes (3 reference genes and 3 target genes) and measured their efficiency in 4 CLEAR samples that were used in generating RA datasets in a dilution series. A simple regression of C_t_ values on the log2 transformed dilution factors showed that the amplification efficiencies are quite different across genes but fairly similar across samples (Table 3) in our experiment. When the target genes are regressed onto the reference genes, the differences in amplification efficiencies resulted in coefficients deviating from 1 (Figure 5).

**Table 3:** **Regression coefficients of C**_**t**_ **values on dilution factors**

**Samples**	**RPS9**	**GAPDH**	**RPL13A**	**IFNGR1**	**IRF1**	**LY96**
X5028	−1.133	−1.224	−1.062	−1.541	−1.082	−1.249
X5030	−1.078	−1.229	−1.072	−1.63	−1.081	−1.194
X7057	−1.094	−1.218	−1.088	−1.583	−1.072	−1.145
X7062	−1.09	−1.229	−1.081	−1.621	−1.091	−1.189

Dilution factors were log_2_ transformed. The ideal 100% efficiency corresponds to coefficient of −1.

### Impact on differential expression analyses

**Figure 6:**
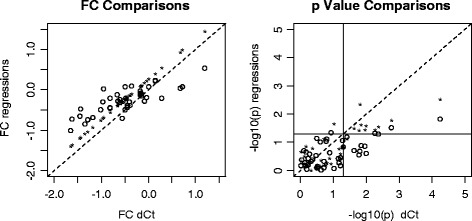
**Fold change and p value comparisons between ΔC**_**t**_ **and regression-based normalization methods.** FC, fold change. For the ΔC_t_ method, the normalization factor is the mean C_t_ of the 5 reference genes; For the regression-based method, normalization factors are the multiple regressions of the mean C_t_ values of target genes (*) or the C_t_ values of each target gene (o) against the mean C_t_ of reference genes. The dashed line is the identity line. The vertical and horizontal lines in the right panel mark the significance level of nominal p value 0.05.

To assess the impact of the ΔC_t_ bias on differential gene expression analysis, we compared the regression-based strategies (C_t_ mean regression and per-gene regression) with the conventional ΔC_t_ method for difference in expression fold change and p values using the RA-SAB dataset. For convenience, we only examined the 42 target genes without any undetectable values from a subset of the samples with the most extreme differences in clinical phenotype (RA subjects with early disease and significant radiographic damage, and controls without autoimmune disease) using the Wilcoxon Rank Sum test. The fold change estimations (group mean C_t_ differences) from the three methods are highly correlated (left panel of Figure 6). Those from the C_t_ mean regression are simply shifted by a constant from the ΔC_t_ method. On the contrary, the per-gene regression method generated smaller fold changes than the other two methods (above the identity line for the down-regulated genes and below the identity line for the up-regulated genes in the left panel of Figure 5). When p values were compared, the ΔC_t_ and C_t_ mean regression methods identified almost exactly the same genes as being differentially expressed between the two groups of subjects*;* however the p values tended to be larger from the C_t_ mean regression (right panel of Figure 6). In contrast, the per-gene regression method identified fewer significantly differentially expressed genes and the p values were larger than those from the other two methods.

We conducted some simple simulation studies to compare the fold change estimates and false/true positive rates between ΔC_t_ normalization and per-gene regression normalization. Our results showed that the per-gene regression normalization increase the precision of fold change estimates (Additional file 1: Figure S2) and the power for detecting differential expressions especially when the regression coefficient is far from 1 and the variation is not too large. The false positive rate of the regression normalization is well controlled around the expected level while that of the ΔC_t_ normalization is inflated when there is a mean C_t_ difference between the comparing groups for the control gene (Additional file 2: Table S3 and Additional file 2: Table S4). The inflation of false positive rate from ΔC_t_ normalization enlarges along the decrease of target gene C_t_ variance and the increase of sample size.

## Discussion

Our study showed that even with a universally constant reference gene, the ΔC_t_ method tends to introduce large bias. Although the C_t_ values of the target genes are positively correlated with the reference gene, the regression coefficients are often substantially different from 1. We believe that a more appropriate method is to estimate the coefficient using regression and then subtract the reference gene C_t_ values adjusted by the regression coefficient.

Using three target genes and three reference genes as example, we demonstrated that the RT-qPCR amplification efficiencies are different among genes, which results in the deviation of the regression coefficients from 1 for some combinations of target and reference genes. Under ideal conditions, all primers/probes pairs should have amplification efficiency at close to 100% (http://www3.appliedbiosystems.com/cms/groups/mcb_marketing/documents/generaldocuments/cms_040377.pdf). Otherwise, the amplification efficiency should be estimated [23-25] and incorporated into the normalization procedure. Unfortunately, dilution curves or amplification dynamics for estimating the amplification efficiency of each gene is not a pragmatic method in RT-qPCR experiments. Given the cost of low density RT-qPCR arrays, it is even less practical to run dilution curves. Therefore, a simple remedy is to use regression for each target gene in the normalization instead of direct subtraction of C_t_ values.

Linear regression is a simple and effective way to estimate the normalization coefficients. However, one potential downside is that it can be easily affected by outlier data points. In our analysis, we removed outlier data points before normalization to avoid this problem. An alternative way is to apply a robust regression to combine these two steps together. Attention is also needed when combining RT-qPCR datasets. When individual datasets are normalized separately, the regression coefficients and intercepts can be different. If this is the case, the normalized data based on different regression coefficients will still have potential mean differences, which needs to be adjusted before combining the datasets.

When multiple reference genes are used as controls, they do not always give similar regression coefficients. We showed that using the mean C_t_ values of all reference genes for regression can achieve most of the normalization goal. However multiple regression analysis does a better job at simultaneously removing all dependency on all reference genes. We have found that the coefficients for some reference genes are fairly large while they are close to zero for others (Additional file 2: Table S2). Therefore, using only the reference genes with large coefficients will usually work well. One downside of multiple regression is that when the sample size of the RT-qPCR experiment is small, for example no bigger than the number of reference genes, the multiple regression will over-fit due to the lack of degree of freedom for residuals. In this situation, the number of reference genes used has to be reduced by selecting the best one or using the average. It is important to point out that multiple regression normalization is less stringent than global mean normalization because it does not force the mean C_t_ values of all samples to be the same. It only removes the correlation with reference genes.

When regression-based normalization is conducted for low density RT-qPCR array data, there is the choice of using the mean target C_t_ values of all target genes for a single regression or regression for each target gene on the array. Our results from the RA-SAB dataset showed that the mean regression was just one constant shift from the ΔC_t_ normalization when fold change is concerned. The per-gene regression resulted in more differences due to the regression coefficient differences among genes. The fold changes obtained from the per-gene regression normalization were smaller and p values were larger than those from conventional subtraction normalization. This is likely the result of bias removal. When correlation between normalized target gene C_t_ and control C_t_ is introduced by subtraction normalization, fold change has two components, the true fold change between the two comparing groups and the difference related to the mean control difference. For example, even if two groups have equal mean expressions, the two group means of the normalized ΔC_t_ values will still be different when the data points from the two groups are located in different areas in a panel of Figure 2. The size of this difference depends on the slope of regression and the mean difference of the control gene C_t_ values. Therefore, ΔC_t_ normalization gives larger fold changes, which results in smaller p values. Our simulation results largely confirmed this speculation. Bias related to ΔC_t_ normalization could be one reason for larger fold changes obtained from RT-qPCR than those from other high-throughput technologies, such as microarrays. Given that RT-qPCR has been considered as “gold standard” for quantifying gene expression, the general thoughts about this discrepancy have been that microarray somehow “squashes” the fold changes. Given our findings in this study, an alternative explanation is that RT-qPCR sometimes inflates fold changes due to ΔC_t_ bias. This is consistent with the observations that fold changes from microarray and RNA-Seq have been found to be very similar in some studies [26,27].

One limitation of our regression-based normalization is that it works well when the sample size of the experiment is fairly large, such as our example (n = 60) and the GEO datasets (n ≥ 12). It can be problematic for very small sample sizes, such as just a few. Our simulations showed that the reduction of false positives and gain of power diminishes when total sample size goes down to 10 when variation is large. For RT-qPCR experiments on single or a few genes, dilution series are needed and practical for estimating amplification efficiencies, which can then be taken into account in normalization. For RT-qPCR array experiments with small number of samples, dilution series is less practical due to the cost. In this case, the amplification efficiency can be estimated based on the PCR kinetic curve [24,25]. However, kinetic curves have to be obtained for each gene from the PCR machine, which is not a standard practice of RT-qPCR. If these methods are not applied, investigators need to be aware of the existence of potential bias associated with ΔC_t_ normalization in differential expression. In addition, we recommend use regression-based normalization when a statistically significant correlation between the C_t_ values of target genes and controls is detected; otherwise, the regression-based normalization is not beneficial.

## Conclusions

The ΔC_t_ normalization method often introduces bias due to amplification efficiency differences, which affects the estimation of fold change and the identification of differentially expressed genes. This bias can be effectively corrected by estimating the regression coefficient for each target gene and adjusting their ΔC_t_ values accordingly.

## Methods

### Datasets

#### Rheumatoid arthritis datasets

Two low-density PCR arrays were used to generate gene expression data from peripheral blood cells of patients with rheumatoid arthritis (RA). The first array was the Innate and Adaptive Immune Responses PCR Array from SABiosciences (Frederick, MD), which has 84 genes involved in the host response to bacterial infection and sepsis with 5 reference genes (http://www.sabiosciences.com/rt_pcr_product/HTML/PAHS-052A.html). The second array was the TaqMan Human Immune Array from Applied Biosystems (Foster City, CA), which contains 90 genes involved in stress response, signal transduction, cytokines/receptors, cell surface receptors, oxidoreductase, chemokines, protease, and cell cycle. Six reference genes are included as internal controls (http://tools.lifetechnologies.com/content/sfs/brochures/cms_042394.pdf). 60 RNA samples from peripheral blood (collected in PAXGene tubes) were studied from 40 African-Americans with RA and 20 African-American healthy controls. All patients and controls were from the CLEAR (Consortium for the Longitudinal Evaluation of African-Americans with Rheumatoid Arthritis) Registry [28,29] (http://www.uab.edu/medicine/rheumatology/research/70-clear). This study was approved by the Institutional Review Board (IRB) for Human Use of the University of Alabama at Birmingham (UAB IRB Human Subjects Protocol # X080219016). All participants and controls signed informed consent forms, and all human subject research was in compliance with the Helsinki Declaration. Standard protocols recommended by the manufacturer were used for preparing cDNA, PCR amplification, and quantification. PCR amplification was conducted using the Applied Biosystems Prism 7900HT sequence detection system.

#### Public datasets from Gene Expression Omnibus (GEO)

We selected RT-qPCR array datasets that have large or moderate sample size. Raw data were downloaded for each dataset (Additional file 2: Table S1). The median C_t_ value from the technical replicates of each gene was used for further analyses.

#### PCR amplification efficiency experiment

We selected six genes with three reference genes (GAPDH, RPS9, and RPL13A) and three target genes (IFNGR1, IRF1, and LY96). TaqMan® Assays recommended by the manufacture as most efficient for quantifying gene expression from each gene were purchased from Life Technologies (Grand Island, NY). Seven concentrations (1/16, 1/8, 1/4, ½, 1, 2, and 4 fold of the original cDNA concentration) were used for examining amplification efficiency. The reactions were performed on an Applied Biosystems QuantStudioTM 6 Flex Real-Time PCR System (384-well, 15 uL reaction volume/well). Three technical replicates were conducted for each sample and all samples were on the same plate.

### Analysis methods

RT-qPCR data filtering: The median was used to summarize the three technical replicates from the same sample. For the RA data, we filtered out the C_t_ values that were equal to 40 (undetectable) for downstream analysis. For GEO datasets, C_t_ values of 35, 39, 40, or “undetermined” were filtered out depending on the truncation value of the dataset for non-detection. Outlier C_t_ values were identified for each reference gene as more than 1.5 times of inter quartile range beyond the first and third quartiles. Samples with more than one reference genes deemed as outliers were removed from calculating the regression coefficients to avoid outlier effect. For testing of differential expression between two groups we used the Wilcoxon Rank Sum test after filtering the undetectable samples. The differences of group means were used to represent the fold changes for comparison of normalization methods. The data organization were coducted using Microsoft Excel. Statistical tests and plots for figures were conducted in R (version 3.0.0). All plots were generated using the *plot* function in R.

### Proposed regression-based normalization

After removing undetectable target genes, follow three simple steps for each target gene. 1) Remove samples with outlier control gene expressions. 2) Regress target gene C_t_ values onto a control gene C_t_ values to obtain regression coefficient (*b*) and test for its significance; 3) If *b* is significant, conduct the normalization as C_t__target – *b* x C_t__control. When there are multiple control genes and a large enough sample size, conduct multiple regression with all control genes in the model as dependant variables to estimate their regression coefficients. Perform normalization by subtracting all control gene C_t_ values multiplied by their corresponding regression coefficients.

### Data access

The RA-qPCR array data associated with this study have been submitted to NCBI GEO with accession number GSE64708.

## Additional files

Additional file 1: Figure S1. Shows the dot plots of C_t_ values of individual target genes against the mean C_t_ values of control genes. **Figure S2.** shows the comparison of fold changes estimated from dC_t_ and per-gene regression normalizations in a simulation. Additional file 2: Table S1. Provides a summary of the datasets used in the paper. **Table S2.** gives the multiple regression coefficients for housekeeping genes on C_t_ means of target genes. **Table S3.** shows the simulation results based on equal group means for control gene C_t_ values. **Table S4.** shows the simulation results based on unequal group means for control gene C_t_ values.
