# Supplementary material for: Simple regression for correcting ΔCt bias in RT-qPCR low-density array data normalization
Source: BMC Genomics. 2015 Feb 14;16(1):82. doi: 10.1186/s12864-015-1274-1 (PMC4335788; doi:10.1186/s12864-015-1274-1)
Supplement: Additional file 1: Figure S1. — Shows the dot plots of Ct values of individual target genes against the mean Ct values of control genes. Figure S2. shows the comparison of fold changes estimated from dCt and per-gene regression normalizations in a simulation. [file 12864_2015_1274_MOESM1_ESM.docx]

**Figure S1.** Regression of 42 target genes on to the mean C_t_ of reference genes. Only genes without undetectable values from the RA SAB dataset was used. X-axis is the mean C_t_ values of the reference genes. Y-axis is the C_t_ values of a target gene.

**Figure S2.** Comparison of fold change estimates from dC_t_ and per-gene regression normalizations in a simulation study. The simulation was conducted as described in the **Supp. Table 3** legends. The parameter setting are: sample size (n)=20, regression coefficient (b) = 0.58, and variation (sd) =0.2. The fold change estimates from the regression normalization (FC.rg) are well separated in the three simulated group means, 0, log_2_(1.5) and 1og_2_(2). The estimates from dC_t_ normalization (FC.dCt) have much more variability around the simulated values than those from the per-gene regression normalization (FC.rg).
