# Supplementary material for: Simple regression for correcting ΔCt bias in RT-qPCR low-density array data normalization
Source: BMC Genomics. 2015 Feb 14;16(1):82. doi: 10.1186/s12864-015-1274-1 (PMC4335788; doi:10.1186/s12864-015-1274-1)
Supplement: Additional file 2: Table S1. — Provides a summary of the datasets used in the paper. Table S2. gives the multiple regression coefficients for housekeeping genes on Ct means of target genes. Table S3. shows the simulation results based on equal group means for control gene Ct values. Table S4. shows the simulation results based on unequal group means for control gene Ct values. [file 12864_2015_1274_MOESM2_ESM.docx]

**Table S1**. Dataset summary

| Target Type | Dataset | Array | # of Targets | Internal Controls | # of Samples |
| --- | --- | --- | --- | --- | --- |
| mRNA | RA-ABI | Innate and Adaptive Immune Responses PCR Array from SABiosciences | 90 | GADPH, ACTB. 18S, GUSB, PGK1, TFRC | 60 |
|  | RA- SAB | TaqMan Human Immune Array from Applied Biosystems | 84 | B2M, HPRT1, RPL13A, GADPH, ACTB | 60 |
|  | GSE 11690 GPL 6926 | DNA damage signaling APHS-029BQRT-PCR | 84 | B2M, HPRT1, RPL13A, GADPH, ACTB | 12 |
|  | GSE 11690 GPL 6933 | DNA damage signaling APHS-029BQRT-PCR | 84 | B2M, HPRT1, RPL13A, GADPH, ACTB | 24 |
|  | GSE 15488 GPL 8370 | Human stress &toxicity pathway PAHS-003A | 84 | B2M, HPRT1, RPL13A, GADPH, ACTB | 24 |
| microRNA | GSE 19229 GPL 9732 | Taqman microRNA Low density arrays (TLDA) panel B | 381 | MammU6, RNU44, RNU48 | 28 |
|  | GSE 22264 GPL 10522 | TaqMan Human MicroRNA Array v1.0 | 365 | RNU44, RNU48, RNU6B | 34 |
|  | GSE 39105 GPL 15765 | Life Technologies Human miRNA expression assays 450-plex | 430 | RNU44, RNU48, RNU49 | 36 |
|  | GSE 25968 GPL 11239 | TaqMan array miRNA card-A | 382 | RNU44, RNU 48 | 49 |

**Table S2.** Multiple regression coefficients for housekeeping genes on C_t_ means of target genes

| Internal Controls | GSE15488 GPL8370 | GSE 11690 GPL6933 | GSE11690 GPL6926 | RA-SAB | RA-ABI |
| --- | --- | --- | --- | --- | --- |
| B2M | 0.24368 | -0.22088 | 0.37348 | 0. 0840 | NA |
| HPRT1 | 0.06376 | 0.05677 | -0.02201 | -0. 0083 | NA |
| RPL13A | 0.64431 | -0.33414 | -0.02579 | 0. 0049 | NA |
| GAPDH | 0.37556 | 0.04901 | -0.13441 | 0. 3076 | -0.1515 |
| ACTB | -0.34222 | 1.27660 | 0.96148 | 0. 3237 | 0.2166 |
| 18S | NA | NA | NA | NA | -0.0318 |
| GUSB | NA | NA | NA | NA | 0.4225 |
| PGK1 | NA | NA | NA | NA | 0.1311 |
| TFRC | NA | NA | NA | NA | 0.0098 |

NA, not assayed.

**Table S3.** Simulation results from equal group means of control gene C_t_ values

| False positive rate (type I error) | | | |  |  |  |  |  |  |
| --- | --- | --- | --- | --- | --- | --- | --- | --- | --- |
|  |  | b=0.5 | | b=0.7 | | b=0.9 | | b=1.2 | |
|  |  | dCt | rg | dCt | rg | dCt | rg | dCt | rg |
| n=10 | sd=0.2 | 0.055 | 0.06 | 0.04 | 0.0375 | 0.055 | 0.06 | 0.035 | 0.035 |
|  | sd=0.4 | 0.055 | 0.0475 | 0.0625 | 0.0425 | 0.0375 | 0.045 | 0.04 | 0.055 |
|  | sd=0.6 | 0.0425 | 0.045 | 0.0275 | 0.0325 | 0.0475 | 0.0525 | 0.0325 | 0.04 |
|  | sd=0.8 | 0.03 | 0.045 | 0.035 | 0.0425 | 0.0525 | 0.045 | 0.05 | 0.0325 |
| n=20 | sd=0.2 | 0.0475 | 0.0625 | 0.045 | 0.05 | 0.0625 | 0.0575 | 0.03 | 0.03 |
|  | sd=0.4 | 0.065 | 0.06 | 0.0575 | 0.05 | 0.05 | 0.0375 | 0.0725 | 0.05 |
|  | sd=0.6 | 0.05 | 0.0225 | 0.0575 | 0.06 | 0.0575 | 0.0575 | 0.0375 | 0.03 |
|  | sd=0.8 | 0.0525 | 0.045 | 0.06 | 0.0625 | 0.05 | 0.05 | 0.0575 | 0.055 |
| n=30 | sd=0.2 | 0.05 | 0.0575 | 0.0575 | 0.0675 | 0.0475 | 0.05 | 0.0575 | 0.06 |
|  | sd=0.4 | 0.0475 | 0.055 | 0.055 | 0.0475 | 0.045 | 0.0525 | 0.0325 | 0.04 |
|  | sd=0.6 | 0.035 | 0.035 | 0.0625 | 0.035 | 0.045 | 0.0375 | 0.0525 | 0.0575 |
|  | sd=0.8 | 0.0425 | 0.0425 | 0.0525 | 0.0575 | 0.0425 | 0.0525 | 0.04 | 0.025 |
| n=40 | sd=0.2 | 0.05 | 0.0525 | 0.0575 | 0.055 | 0.0525 | 0.055 | 0.0425 | 0.0375 |
|  | sd=0.4 | 0.055 | 0.05 | 0.04 | 0.06 | 0.055 | 0.0725 | 0.0575 | 0.0575 |
|  | sd=0.6 | 0.0475 | 0.045 | 0.055 | 0.035 | 0.045 | 0.04 | 0.06 | 0.0625 |
|  | sd=0.8 | 0.0375 | 0.055 | 0.0475 | 0.07 | 0.05 | 0.0525 | 0.06 | 0.06 |
|  |  |  |  |  |  |  |  |  |  |
| True positive rate (power) for fold change of 1.5. | | | | |  |  |  |  |  |
|  |  | b=0.5 | | b=0.7 | | b=0.9 | | b=1.2 | |
|  |  | dCt | rg | dCt | rg | dCt | rg | dCt | rg |
| n=10 | sd=0.2 | 0.2975 | 0.905 | 0.62 | 0.89 | 0.93 | 0.9175 | 0.7925 | 0.92 |
|  | sd=0.4 | 0.25 | 0.4625 | 0.3525 | 0.4725 | 0.4675 | 0.43 | 0.455 | 0.4375 |
|  | sd=0.6 | 0.155 | 0.215 | 0.23 | 0.2325 | 0.225 | 0.21 | 0.2175 | 0.2125 |
|  | sd=0.8 | 0.1125 | 0.145 | 0.145 | 0.13 | 0.1775 | 0.145 | 0.1725 | 0.165 |
| n=20 | sd=0.2 | 0.6025 | 1 | 0.925 | 1 | 1 | 1 | 0.995 | 1 |
|  | sd=0.4 | 0.4775 | 0.83 | 0.7175 | 0.8575 | 0.83 | 0.835 | 0.805 | 0.8675 |
|  | sd=0.6 | 0.3175 | 0.5225 | 0.4125 | 0.4825 | 0.5075 | 0.4975 | 0.5 | 0.5 |
|  | sd=0.8 | 0.26 | 0.335 | 0.26 | 0.2875 | 0.3575 | 0.3225 | 0.3425 | 0.3475 |
| n=30 | sd=0.2 | 0.8275 | 1 | 0.9925 | 1 | 1 | 1 | 1 | 1 |
|  | sd=0.4 | 0.6825 | 0.9525 | 0.875 | 0.955 | 0.95 | 0.955 | 0.95 | 0.9875 |
|  | sd=0.6 | 0.505 | 0.735 | 0.6175 | 0.705 | 0.675 | 0.635 | 0.6875 | 0.695 |
|  | sd=0.8 | 0.3125 | 0.4725 | 0.4575 | 0.5 | 0.445 | 0.4375 | 0.465 | 0.47 |
| n=40 | sd=0.2 | 0.9025 | 1 | 0.995 | 1 | 1 | 1 | 1 | 1 |
|  | sd=0.4 | 0.8375 | 0.99 | 0.9375 | 0.985 | 0.9925 | 0.99 | 0.98 | 0.9925 |
|  | sd=0.6 | 0.5925 | 0.8375 | 0.78 | 0.82 | 0.85 | 0.8475 | 0.86 | 0.88 |
|  | sd=0.8 | 0.4875 | 0.61 | 0.52 | 0.5825 | 0.635 | 0.63 | 0.58 | 0.6075 |
|  |  |  |  |  |  |  |  |  |  |
| True positive rate (power) for fold change of 2. | | | | |  |  |  |  |  |
|  |  | b=0.5 | | b=0.7 | | b=0.9 | | b=1.2 | |
|  |  | dCt | rg | dCt | rg | dCt | rg | dCt | rg |
| n=10 | sd=0.2 | 0.675 | 0.9775 | 0.9525 | 0.975 | 1 | 0.975 | 0.9975 | 0.98 |
|  | sd=0.4 | 0.515 | 0.8025 | 0.7525 | 0.8375 | 0.8925 | 0.8325 | 0.84 | 0.815 |
|  | sd=0.6 | 0.39 | 0.5225 | 0.52 | 0.5225 | 0.615 | 0.54 | 0.5525 | 0.5375 |
|  | sd=0.8 | 0.2575 | 0.3375 | 0.3575 | 0.3325 | 0.3775 | 0.31 | 0.3725 | 0.3225 |
| n=20 | sd=0.2 | 0.9725 | 1 | 1 | 1 | 1 | 1 | 1 | 1 |
|  | sd=0.4 | 0.9175 | 0.9975 | 0.9875 | 0.9975 | 1 | 0.9975 | 0.9975 | 1 |
|  | sd=0.6 | 0.78 | 0.9475 | 0.88 | 0.9175 | 0.925 | 0.905 | 0.92 | 0.92 |
|  | sd=0.8 | 0.6375 | 0.72 | 0.7325 | 0.7225 | 0.7725 | 0.74 | 0.74 | 0.715 |
| n=30 | sd=0.2 | 0.9975 | 1 | 1 | 1 | 1 | 1 | 1 | 1 |
|  | sd=0.4 | 0.985 | 1 | 1 | 1 | 1 | 1 | 1 | 1 |
|  | sd=0.6 | 0.925 | 0.9825 | 0.965 | 0.98 | 0.9925 | 0.9875 | 0.985 | 0.9875 |
|  | sd=0.8 | 0.825 | 0.895 | 0.88 | 0.9 | 0.895 | 0.8725 | 0.8825 | 0.8875 |
| n=40 | sd=0.2 | 1 | 1 | 1 | 1 | 1 | 1 | 1 | 1 |
|  | sd=0.4 | 0.9975 | 1 | 1 | 1 | 1 | 1 | 1 | 1 |
|  | sd=0.6 | 0.9775 | 0.9975 | 0.9975 | 1 | 1 | 0.9975 | 0.9975 | 1 |
|  | sd=0.8 | 0.9025 | 0.9675 | 0.935 | 0.95 | 0.965 | 0.9625 | 0.9625 | 0.9625 |

Simulations for target gene C_t_ values were set up for two-group comparisons with sample size, n/2, for each group using formula$y=a+bx+\varepsilon$. The control gene C_t_ values, *x*, were randomly drawn from a normal distribution of N(25, 1). The regression coefficients, *b*, were set from 0.5 to 1.2. The residual error was randomly drawn from normal distribution (0, sd), where sd was set based on the estimations from the RA dataset. The intercept, *a*, was set as 10. A total of 1200 genes were simulated. The first 400 genes had same group means for estimating the false positive rate. The second 400 genes had group mean difference of log_2_(1.5), fold change of 1.5. The third 400 genes had group mean difference of log_2_(2), fold change of 2. The data were normalized with conventional ΔC_t_ (dC_t_) method and per-gene regression (rg) method. Two-group t test was used to test for difference between the two groups for each gene. Nominal p value of 0.05 was used to select significant genes. The results showed the false positive rate were similar between the two normalization methods, while the power is higher from the per-gene regression normalization, especially when the simulated regression coefficient *b* is far from 1 and variation sd is small. When variation is large and sample size is small (such as n=10), the improvement is limited due to the lack of accuracy in estimating the regression coefficients.

**Table S4.** Simulation results from unequal group means of control gene C_t_ values

| False positive rate (type I error) | | | |  |  |  |  |  |  |
| --- | --- | --- | --- | --- | --- | --- | --- | --- | --- |
|  |  | b=0.5 | | b=0.7 | | b=0.9 | | b=1.2 | |
|  |  | dCt | rg | dCt | rg | dCt | rg | dCt | rg |
| n=10 | sd=0.2 | 0.1 | 0.025 | 0.065 | 0.0475 | 0.055 | 0.04 | 0.06 | 0.045 |
|  | sd=0.4 | 0.0675 | 0.025 | 0.0575 | 0.035 | 0.0425 | 0.03 | 0.0725 | 0.0425 |
|  | sd=0.6 | 0.0775 | 0.045 | 0.075 | 0.0525 | 0.045 | 0.0375 | 0.0425 | 0.0325 |
|  | sd=0.8 | 0.0325 | 0.03 | 0.0425 | 0.015 | 0.0325 | 0.04 | 0.0475 | 0.04 |
| n=20 | sd=0.2 | 0.1625 | 0.05 | 0.145 | 0.035 | 0.055 | 0.025 | 0.1175 | 0.045 |
|  | sd=0.4 | 0.125 | 0.025 | 0.11 | 0.045 | 0.05 | 0.0575 | 0.0675 | 0.03 |
|  | sd=0.6 | 0.0875 | 0.04 | 0.0625 | 0.035 | 0.075 | 0.0575 | 0.0525 | 0.0475 |
|  | sd=0.8 | 0.0975 | 0.0675 | 0.0625 | 0.0375 | 0.0575 | 0.0475 | 0.0575 | 0.0325 |
| n=30 | sd=0.2 | 0.225 | 0.0325 | 0.18 | 0.04 | 0.1025 | 0.0475 | 0.165 | 0.065 |
|  | sd=0.4 | 0.17 | 0.065 | 0.145 | 0.0675 | 0.065 | 0.0475 | 0.0975 | 0.0675 |
|  | sd=0.6 | 0.17 | 0.04 | 0.0925 | 0.0525 | 0.05 | 0.0375 | 0.06 | 0.0325 |
|  | sd=0.8 | 0.115 | 0.0275 | 0.0575 | 0.0275 | 0.05 | 0.035 | 0.0575 | 0.0475 |
| n=40 | sd=0.2 | 0.3 | 0.0475 | 0.2375 | 0.0475 | 0.095 | 0.0475 | 0.19 | 0.075 |
|  | sd=0.4 | 0.2125 | 0.05 | 0.1525 | 0.0275 | 0.06 | 0.0325 | 0.105 | 0.045 |
|  | sd=0.6 | 0.19 | 0.0475 | 0.0975 | 0.0475 | 0.06 | 0.055 | 0.1 | 0.0375 |
|  | sd=0.8 | 0.1175 | 0.05 | 0.095 | 0.03 | 0.0525 | 0.0425 | 0.0725 | 0.04 |
|  |  |  |  |  |  |  |  |  |  |
| True positive rate (power) for fold change of 1.5. | | | | |  |  |  |  |  |
|  |  | b=0.5 | | b=0.7 | | b=0.9 | | b=1.2 | |
|  |  | dCt | rg | dCt | rg | dCt | rg | dCt | rg |
| n=10 | sd=0.2 | 0.2975 | 0.905 | 0.62 | 0.89 | 0.93 | 0.9175 | 0.7925 | 0.92 |
|  | sd=0.4 | 0.25 | 0.4625 | 0.3525 | 0.4725 | 0.4675 | 0.43 | 0.455 | 0.4375 |
|  | sd=0.6 | 0.155 | 0.215 | 0.23 | 0.2325 | 0.225 | 0.21 | 0.2175 | 0.2125 |
|  | sd=0.8 | 0.1125 | 0.145 | 0.145 | 0.13 | 0.1775 | 0.145 | 0.1725 | 0.165 |
| n=20 | sd=0.2 | 0.6025 | 1 | 0.925 | 1 | 1 | 1 | 0.995 | 1 |
|  | sd=0.4 | 0.4775 | 0.83 | 0.7175 | 0.8575 | 0.83 | 0.835 | 0.805 | 0.8675 |
|  | sd=0.6 | 0.3175 | 0.5225 | 0.4125 | 0.4825 | 0.5075 | 0.4975 | 0.5 | 0.5 |
|  | sd=0.8 | 0.26 | 0.335 | 0.26 | 0.2875 | 0.3575 | 0.3225 | 0.3425 | 0.3475 |
| n=30 | sd=0.2 | 0.8275 | 1 | 0.9925 | 1 | 1 | 1 | 1 | 1 |
|  | sd=0.4 | 0.6825 | 0.9525 | 0.875 | 0.955 | 0.95 | 0.955 | 0.95 | 0.9875 |
|  | sd=0.6 | 0.505 | 0.735 | 0.6175 | 0.705 | 0.675 | 0.635 | 0.6875 | 0.695 |
|  | sd=0.8 | 0.3125 | 0.4725 | 0.4575 | 0.5 | 0.445 | 0.4375 | 0.465 | 0.47 |
| n=40 | sd=0.2 | 0.9025 | 1 | 0.995 | 1 | 1 | 1 | 1 | 1 |
|  | sd=0.4 | 0.8375 | 0.99 | 0.9375 | 0.985 | 0.9925 | 0.99 | 0.98 | 0.9925 |
|  | sd=0.6 | 0.5925 | 0.8375 | 0.78 | 0.82 | 0.85 | 0.8475 | 0.86 | 0.88 |
|  | sd=0.8 | 0.4875 | 0.61 | 0.52 | 0.5825 | 0.635 | 0.63 | 0.58 | 0.6075 |
|  |  |  |  |  |  |  |  |  |  |
| True positive rate (power) for fold change of 2. | | | | |  |  |  |  |  |
|  |  | b=0.5 | | b=0.7 | | b=0.9 | | b=1.2 | |
|  |  | dCt | rg | dCt | rg | dCt | rg | dCt | rg |
| n=10 | sd=0.2 | 0.5 | 0.95 | 0.89 | 0.9325 | 1 | 0.9325 | 1 | 0.94 |
|  | sd=0.4 | 0.3425 | 0.7925 | 0.6325 | 0.73 | 0.8875 | 0.725 | 0.9425 | 0.7475 |
|  | sd=0.6 | 0.2525 | 0.45 | 0.43 | 0.4825 | 0.545 | 0.465 | 0.6475 | 0.475 |
|  | sd=0.8 | 0.195 | 0.2875 | 0.3 | 0.34 | 0.355 | 0.2875 | 0.4 | 0.32 |
| n=20 | sd=0.2 | 0.85 | 1 | 1 | 1 | 1 | 1 | 1 | 1 |
|  | sd=0.4 | 0.6575 | 0.9825 | 0.95 | 0.9925 | 0.9975 | 0.9925 | 1 | 0.985 |
|  | sd=0.6 | 0.535 | 0.86 | 0.7375 | 0.875 | 0.9025 | 0.87 | 0.9675 | 0.8475 |
|  | sd=0.8 | 0.3775 | 0.63 | 0.59 | 0.6425 | 0.685 | 0.6175 | 0.815 | 0.665 |
| n=30 | sd=0.2 | 0.955 | 1 | 1 | 1 | 1 | 1 | 1 | 1 |
|  | sd=0.4 | 0.8925 | 1 | 0.99 | 0.9975 | 1 | 1 | 1 | 1 |
|  | sd=0.6 | 0.725 | 0.9875 | 0.91 | 0.985 | 0.9875 | 0.97 | 0.9975 | 0.9825 |
|  | sd=0.8 | 0.5975 | 0.8775 | 0.75 | 0.8325 | 0.8775 | 0.8575 | 0.925 | 0.8475 |
| n=40 | sd=0.2 | 0.99 | 1 | 1 | 1 | 1 | 1 | 1 | 1 |
|  | sd=0.4 | 0.945 | 1 | 1 | 1 | 1 | 1 | 1 | 1 |
|  | sd=0.6 | 0.845 | 0.9975 | 0.9475 | 0.9925 | 0.995 | 0.9975 | 1 | 0.995 |
|  | sd=0.8 | 0.6725 | 0.945 | 0.8425 | 0.9225 | 0.9425 | 0.9475 | 0.9875 | 0.9475 |

Everything is the same as for Supplementary Table 4 except that the mean C_t_ values of the control gene have 0.5 standard deviation difference, with first group drawn from normal distribution N(25,1) and second group drawn from N(25.5, 1). The false positive rate from per-gene regression (rg) normalization is around the nominal 0.05 level, but that of the ΔC_t_ normalization is inflated. The degree of inflation increases as the variation decreases and the sample size increases.
